# Tumor-Derived Exosomal miRNAs as Diagnostic Biomarkers in Non-Small Cell Lung Cancer

**DOI:** 10.3389/fonc.2020.560025

**Published:** 2020-10-14

**Authors:** Zhijun Zhang, Youyong Tang, Xingguo Song, Li Xie, Shuping Zhao, Xianrang Song

**Affiliations:** ^1^Department of Clinical Laboratory, Taian City Central Hospital, Taian, China; ^2^Department of Clinical Laboratory, Shandong Cancer Hospital and Institute, Shandong First Medical University and Shandong Academy of Medical Sciences, Jinan, China; ^3^Shandong Provincial Key Laboratory of Radiation Oncology, Shandong Cancer Hospital and Institute, Shandong First Medical University and Shandong Academy of Medical Sciences, Jinan, China

**Keywords:** non-small cell lung cancer, serum, exosomes, MiR-5684, MiR-125b-5p, biomarker, diagnosis

## Abstract

**Background:**

Delayed diagnosis is the main obstacle to improve prognosis of non-small cell lung cancer (NSCLC). Novel biomarkers for the diagnosis of NSCLC are urgently needed. This study aimed to identify the specific exosomal miRNAs with diagnostic and prognostic potential in NSCLC patients.

**Materials and Methods:**

Transmission electron microscopy (TEM), qNano and western blots were used to characterize the exosomes isolated from the serum of NSCLC patients (n=330) and healthy donors (n=312) by ultracentrifugation. Exosomal miRNAs were profiled by miRNA microarrays and verified by quantitative PCR (qPCR). The diagnostic accuracy was determined by receiver operating characteristic (ROC) analysis.

**Results:**

A total of differential 22 miRNAs were screened out based on P < 0.05 and fold difference>2.0 by miRNA microarrays, among which, exosomal miR-5684 and miR-125b-5p were significantly down-regulated in NSCLC patients compared to healthy donors, processing favorable diagnostic efficiency for (early) NSCLC. Importantly, the exosomal miR-125b-5p were associated with metastasis (P < 0.0001), chemotherapeutic effect (P=0.007) and survival (P=0.008).

**Conclusion:**

Exosomal miR-5684 and miR-125b-5p levels are significantly down-regulated in NSCLC patients, and serve as the promising diagnostic and prognostic biomarkers for NSCLC.

## Introduction

Non-small cell lung cancer (NSCLC), the most common type of lung cancer which accounts for about 80% of all lung cancer, is the leading cause of cancer-related deaths worldwide (1). Its high mortality rate is not changed even with recent advances in cancer treatment, largely because approximately two-thirds of patients present with metastatic tumors at the time of diagnosis (2). Therefore, there is an urgent need for more reliable biomarkers for the early diagnosis of NSCLC and the monitoring of its progression and response to anti-cancer therapy.

MicroRNAs (miRNAs), the short (19–24 nt) non-coding RNAs, post-transcriptionally regulate gene expression by directly binding to the 3’ untranslated regions (UTRs) of complementary messenger RNA targets (3, 4). Dysregulation of miRNAs expression plays critical roles in the development and progression of different cancers by affecting multiple cellular processes including cell proliferation, apoptosis, survival, invasion, metastasis, and chemotherapeutic resistance (5, 6). MiRNAs can be stably present in the blood called circulating miRNAs in the form of Ago2-miRNA protein complexes to protect against RNase degradation and multiple freeze-thaw cycles (7). Therefore, the differentially expressed circulating miRNAs between lung cancer patients and healthy humans can be used as novel biomarkers for diagnosis, serving the non-invasive biomarkers for NSCLC.

Furthermore, these miRNAs have also been identified in the exosome of plasma and serum in a remarkably stable form and are protected from endogenous RNase activity (7). Exosomes are small (30–150 nm diameter) membrane-bound vesicles that are released by all cell types into body fluids such as saliva, urine, plasma and malignant effusions (8). They can transfer functional proteins, mRNAs, miRNAs and other bioactive molecules to receipt cells, thus playing an important role in intercellular communication (9, 10). Numerous studies have shown that miRNA isolated from circulating exosomes in NSCLC patients mirrors the miRNA pattern expressed in NSCLC tissue (11). Besides, almost all cell types can secrete exosomes under normal or stressful conditions, and cancer cells in particular are known to secrete more exosomes than normal cells of the same organ type (12). All these empower them useful to develop highly sensitive diagnostic strategies for rapid and non-invasive monitoring of the pathological conditions of cancer patients (13) although only a few paper regard NSCLC. For example, it has been reported that exosomal miR-146a-5p, as well as miR-23b-3p, miR-10b-5p, and miR-21-5p act as prognostic biomarkers for NSCLC patients (14, 15).

In the current study, we aimed to investigate the diagnostic and prognostic role of exosomal miRNAs in NSCLC. We screened out exosomal miR-5684 and miR-125b-5p using microarray and validated their expression in a large cohort, followed by the analysis for their diagnostic efficiency and predicting chemotherapy response assessment, thus providing the evidence of exosomal miR-5684 and miR-125b-5p as biomarkers for NSCLC.

## Materials and Methods

### Patients and Healthy Donors

Total 330 NSCLC patients and 312 healthy donors between January 2019 and July 2019 at the Shandong Cancer Hospital and Institute were enrolled in this study. Written informed consent was obtained from all participants. Tumor staging was estimated according to the Eighth Edition AJCC Cancer Staging Manual (16). All patients didn’t receive any anti-tumor treatment before peripheral blood collection, or suffer any other endocrine, immune, or metabolic diseases. The healthy donors did not present any disease. Patient characteristics are summarized in **Tables 1** and **2**. In addition, TCGA miRNA-Seq data of NSCLC patients (BCGSC miRNA Profiling) and the corresponding clinical data (TCGA, http://cance rgeno me.nih.gov) including age, gender, TNM stage, histopathology, survival etc. were downloaded.

**Table 1:** Characteristics of NSCLC patients for differentially expressed serum exosomal miR-5684.

Characteristics		No. case	Median	*P*-value
Age (y)	≤62	178	5.0194	0.450
	>62	147	4.9296	
Gender	Male	195	5.0727	0.051
	Female	130	4.8380	
Smoking	Yes	172	5.0590	0.150
	No	153	4.8886	
Drinking	Yes	101	4.4369	0.981
	No	216	4.4401	
Pathology diagnosis	AC	225	4.9976	0.983
	SCC	101	4.9949	
Lymph node metastasis	Yes	167	5.0468	0.203
	No	142	4.8952	
TNM staging	I-II	137	4.8539	0.072
	III-IV	174	5.0731	
Distant metastasis	Yes	101	4.9451	0.688
	No	210	4.9958	

AC, adenocarcinoma; SCC, squamous cell carcinoma.

**Table 2:** Characteristics of NSCLC patients for differentially expressed serum exosomal miR-125b-5p.

Characteristics		No. case	Median	*P*-value
Age (y)	≤62	174	4.4270	0.839
	>62	144	4.4526	
Gender	Male	192	4.4422	0.944
	Female	126	4.4331	
Smoking	Yes	168	4.4690	0.607
	No	150	4.4045	
Drinking	Yes	105	5.0184	0.659
	No	219	4.9624	
Pathology diagnosis	AC	220	4.3597	0.485
	SCC	98	4.4527	
Lymph node metastasis	Yes	159	4.6104	**0.0008**
	No	143	4.1816	
TNM staging	I-II	136	4.1536	**0.000**
	III-IV	168	4.6557	
Distant metastasis	Yes	99	4.7659	**0.000**
	No	207	4.2471	

AC, adenocarcinoma; SCC, squamous cell carcinoma.

Bold values: significant difference.

### Isolation of Exosomes

The exosomes were separated by ultracentrifugation as described previously (17). Briefly, the serum was centrifuged at 10,000 g at 4°C for 30 min to remove the cell debris, followed by ultracentrifugation at 100,000 g for 2 h at 4°C (Type 50.4 Ti Rotor; Beckman Coulter) to collect exosomes. The exosome pellets were resuspended in PBS for further analysis.

### Transmission Electron Microscopy (TEM)

The exosomes were placed on a copper grid with a 50 µl drop of 1% glutaraldehyde, and transferred to 100 µl distilled water after 5 min. The grids were left undisturbed for 2 min and stained with 50 µl oxalyl uranyl solution (pH 7) for 5 min, and then placed on a glass dish covered with paraffin film on ice. The grids were rinsed seven times with distilled water for 2 min each and observed using a JEM-1200EX transmission electron microscope operating at 100 kV.

### qNano Assay

The size and particle density of the exosomes were measured by TRPS (Tunable Resistive Pulse Sensing, Izon Science Ltd, Christchurch, New Zealand) according to the manufacturer’s instructions. The density of the exosome preparation was normalized using 1×10^13^ particles/ml calibration beads (18). The data was analyzed using Izon Control Suite software v.3.3.2.2000 (Izon Control Suite version 3.3.2.2001; Izon Science).

### Western Blotting

Twenty µg exosomal protein extracts were separated by 10% SDS-PAGE and transferred onto PVDF membranes (Millipore, Billerica, MA, USA). After blocking with 5% skimmed milk for 2 h, the membranes were incubated overnight with primary antibodies including anti-CD9, anti-GM130, anti-CD54 and anti-TSG101 (CST, Danvers, United State) at 4°C and then with the HRP-conjugated secondary antibody at room temperature for 1 h. The protein bands were visualized using ECL blot detection reagent (P0018; Beyotime, Shanghai, China).

### Microarray Analysis

Exosomes were obtained from 5 ml peripheral blood from 1 healthy and 2 NSCLC patients and subjected to microarray. The samples were hybridized onto a miRCURYTM LNA array (v.19.0) after the exosomal RNAs were labeled by the miRCURY Hy3/Hy5 power labeling kit (Vedbaek, Denmark). The expression data were normalized using the miRNAs with intensities ≥30. The significantly differential miRNAs were obtained using the criteria of fold change ≥2.0. MiRNA expression levels between the samples were distinguished by hierarchical clustering.

### RNA Isolation and Quantitative Real-Time PCR (qRT-PCR)

Exosomal RNA was extracted using the TRIzol reagent (Thermo Fisher Scientific, Carlsbad, USA), and reverse transcribed to cDNA using Mix-X miRNA first-strand synthesis kit (TaKaRa Bio, Nojihigashi, Kusatsu, Japan). qPCR was performed using TB-Green Premix Ex Taq II reagent (Takara Bio) on the LC480 (Roche Diagnostics, Germany). The relative gene expression levels were evaluated by the ΔCt method (Ct^miRNA^-Ct^U6^) as previously described (19). U6 was used as an internal control (20). Each sample was analyzed in duplicates. The primers were as follows: Has-miR-5684: 5´-CCCTAGCCAGAGCAACAGAAA-3’; Has-miR-125b-5p: 5´-TCCCTGAGACCCTAACTTGTGA-3’; U6: 5’-TGGAACGCTTCACGAATTTGC-G -3’, and 5’-GGAACGATACAGAGAAGATTAGC -3’.

### Statistical Analysis

Statistical analysis was performed using GraphPad Prism 6.0 (GraphPad Software, San Diego, CA, USA) and SPSS 22.0 (IBM, Ehningen, Germany) software. The Kolmogorov–Smirnov test was carried out to check the normality of the distribution. The normally distributed numeric variables were evaluated by parametric test, while non-normally distributed variables were analyzed by Mann–Whitney test; One-way ANOVA test or Kruskal-Wallis H test was used in comparisons among more than two groups. Receiver operating characteristic (ROC) curves with the corresponding C statistics (area under the curve, AUC), based on the logistic models, were used to determine the corresponding cutoff points with the pathological diagnosis. Data were shown as median ± SD. The overall survival (OS) of the high- and low-expression groups were compared by the Kaplan-Meier method and log-rank test. The univariable and multivariable Cox analysis was performed to test risk score. P<0.05 was considered statistically significant, all tests were set as double-tailed.

## Results

### Identification of Isolated Serum Exosomes

**Figure 1:**
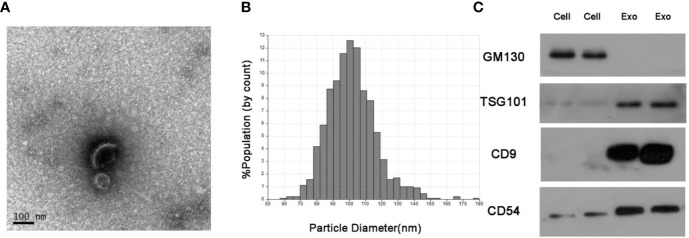
Identification of isolated serum exosomes. **(A)** Representative TEM images of exosomes isolated from the sera of NSCLC patients. **(B)** Size distribution of exosomes ranging from 50–150 nm as analyzed by qNano system. **(C)** Immunoblots showing expression levels of the exosomal proteins CD9, CD54 and TSG101.

The serum exosomes isolated from NSCLC patients and healthy donors were characterized by TEM, qNano and western blots. As shown in **Figures 1A, B**, the exosomes had the typical oval shape and measured 50–150 nm in diameter, and the size range was homogenous. Consistently, the characteristic markers of exosomes including CD9, CD54 and TSG101 were enriched in the exosome but not in the whole cell extracts, whereas GM130 (the negative control) was not detected in the exosomal protein extract (**Figure 1C**).

### Exosomal miR-5684 and miR-125b-5p Are Markedly Downregulated in NSCLC

**Figure 2:**
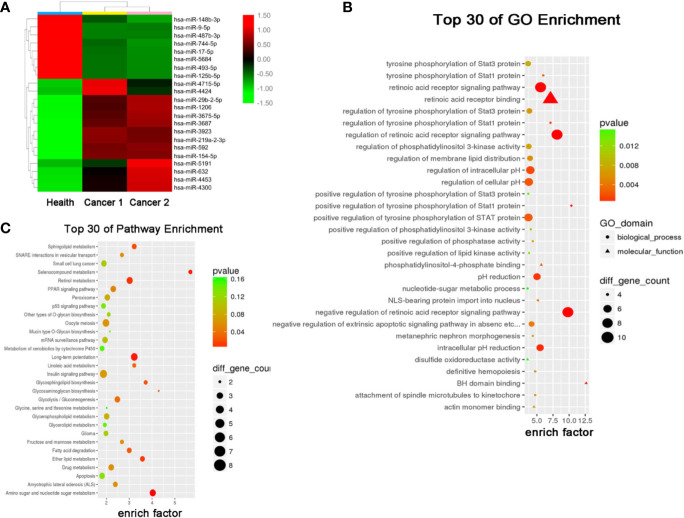
Serum exosomal miRNA profile of the NSCLC patients. **(A)** Cluster analysis of differentially expressed serum exosomal miRNAs between 1 healthy donor and 2 NSCLC patients. Candidate target genes were predicted by **(B)** KEGG enrichment analysis and **(C)** GO analysis.

The results of miRNA profiling for NSCLC have been reported previously (21), from which, we identified 22 differentially expressed miRNAs (DEMs) (8 downregulated and 14 upregulated) in the exosomes of NSCLC patients compared to that of healthy controls (**Figure 2A**). Gene Ontology (GO) and Kyoto Encyclopedia of Genes and Genomes (KEGG) analyses further predicted 30 signaling pathways that were enriched among these DEMs (**Figures 2B, C**), among which retinoic acid receptor binding and long-term potentiation were likely involved in the tumorigenesis and development of NSCLC.

**Figure 3:**
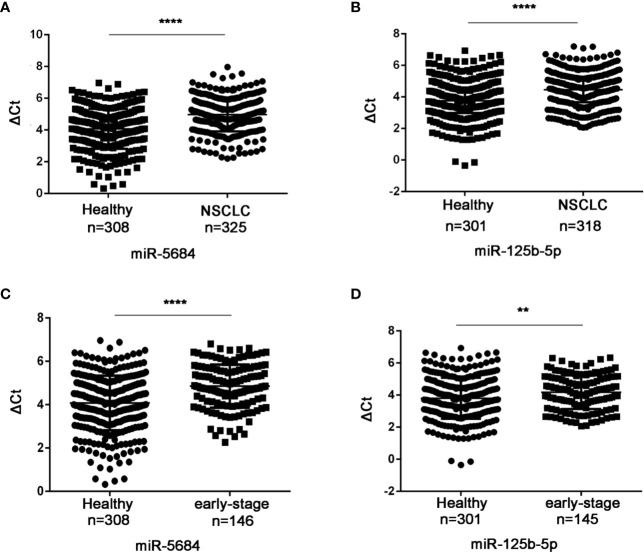
Serum exosomal miR-5684 and miR-125b-5p are potential biomarkers of NSCLC. The expression levels of serum exosomal miR-5684 and miR-125b-5p in **(A)** NSCLC patients and **(B)** healthy donors. The expression levels of serum exosomal miR-5684 and miR-125b-5p in **(C)** early-stage NSCLC patients and **(D)** healthy donors. (****P < 0.0001, **P < 0.01).

Six of these 22 miRNAs were further validated by qRT-PCR in a cohort of 330 NSCLC patients (including 149 at the early-stage, **Table S1**) and 312 healthy donors. As shown in **Figures 3A, B**, miR-5684 and miR-125b-5p were significantly down-regulated in the serum exosomes of the NSCLC patients compared to that of healthy donors (*P* < 0.0001 for both), whereas miR-17-5p, miR-9-5p, miR-148-3p and miR-154-5p did not show any significant difference (**Figure S1**). Furthermore, exosomal miR-5684 and miR-125b-5p levels were markedly lower in the early-stage NSCLC patients compared to healthy donors (*P* < 0.0001 and *P* = 0.002 respectively; **Figures 3C, D**). However, they were irrelated with age, gender, smoking, drinking status and pathological features (**Tables 1** and **2**).

**Figure 4:**
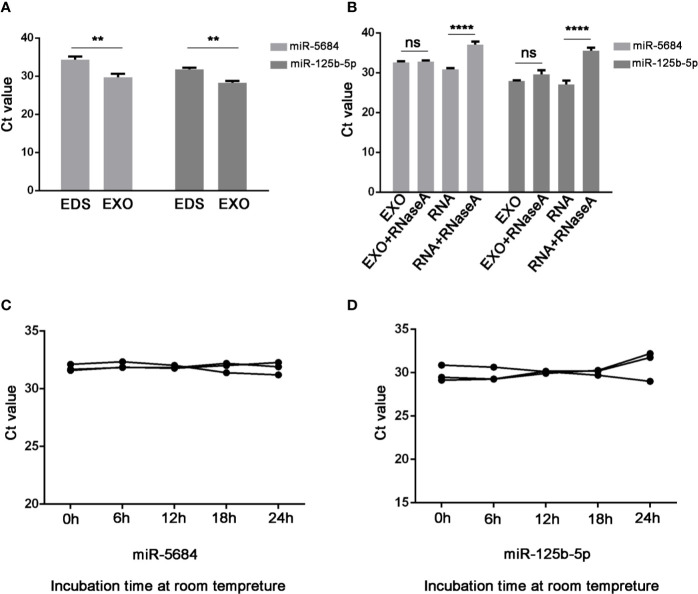
Characteristics of serum exosomal miR-5684 and miR-125b-5p. **(A)** Expression levels of miR-5684 and miR-125b-5p from serum exosomes (EXO) and exosome-depleted supernatant (EDS). **(B)** Expression levels of both miRNAs in exosomes treated with RNase A or in isolated RNA. **(C, D)** Expression levels of the miRNAs in exosomes incubated at room temperature for different durations (**P < 0.01, ****P < 0.0001, ns, not significant).

Notably, both miR-5684 and miR-125b-5p were stably upregulated in the exosomes compared to the exosome-depleted supernatant (EDS) (P=0.003 and P=0.001 respectively; **Figure 4A**), and unaffected by RNase A treatment (**Figure 4B**), or at room temperature for over 24h (**Figures 4C, D**).

### Diagnostic Role of Serum Exosomal miRNAs in NSCLC Patients

**Figure 5:**
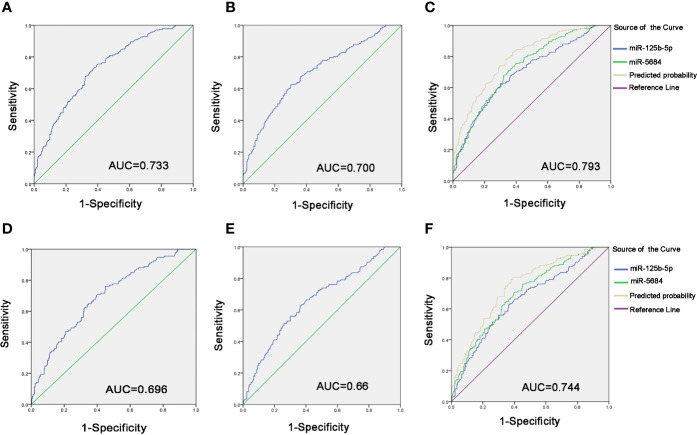
Diagnostic role of serum exosomal miRNA expression levels in NSCLC patients. The AUCs of miR-5684 **(A)**, miR-125b-5p **(B)**, and both **(C)** in NSCLC patients relative to healthy donors. The AUCs of miR-5684 **(D)**, miR-125b-5p **(E)**, and both **(F)** in early-stage NSCLC patients relative to healthy donors.

To evaluate diagnostic performance of exosomal miR-5684 and miR-125b-5p for NSCLC, a receiver-operating characteristic (ROC) curve was calculated (**Tables S2 and S3**). As shown in **Figures 5A, B**, the areas under the curve (AUCs) of exosomal miR-5864 and miR-125b-5p were 0.733 with sensitivity and specificity [95% confidence interval (CI), 0.69–0.775], and 0.700 with sensitivity and specificity (95% CI, 0.655–0.745) with 62.4% sensitivity and 70% specificity compared to the healthy donors. Meanwhile, the diagnostic performance of their combination was also calculated, possessing the AUC of 0.793 (95% CI, 0.755–0.831) with 82.7% sensitivity and 62.1%, indicating exosomal miR-5684 and miR-125b-5p potentially act as the non-invasive circulating biomarkers for lung cancer (**Figure 5C**).

Similarly, when comparing the patients with early stage NSCLC to healthy controls, ROC curves demonstrated favorable diagnostic efficiencies of exosomal miR-5684 and miR-125b-5p, processing AUCs of 0.696 with 76.1% sensitivity and 54.9% specificity, 0.66 with 62.7% sensitivity and 63.6% specificity, respectively (**Figures 5D, E**). Moreover, the diagnostic performance for their combination demonstrated the AUC of 0.744 with a relative sensitivity of 80.6% and a relative specificity of 60.9% (**Figure 5F**).

### The Combination of Serum Exosomal miRNA and Tumor Markers Increases Diagnostic Accuracy of NSCLC

**Figure 6:**
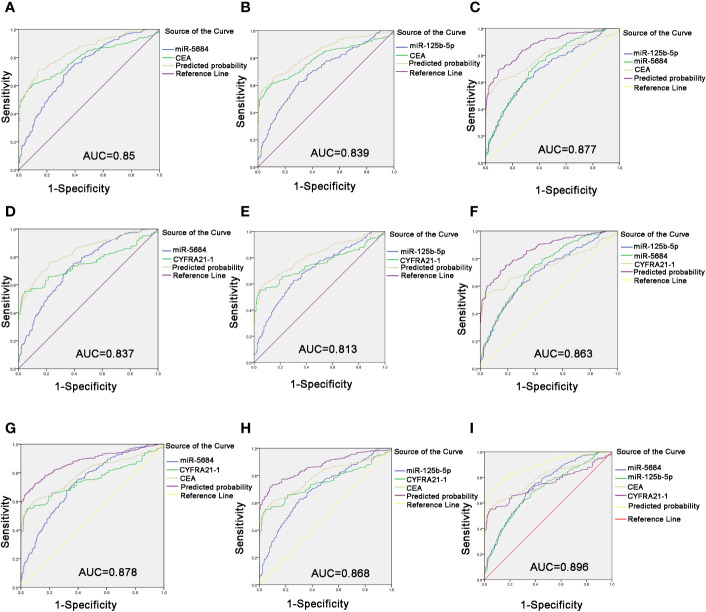
Improved diagnostic capacity of serum exosomal miRNAs combined with established tumor markers in NSCLC patients. The AUCs of CEA combined with miR-5684 **(A)**, miR-125b-5p **(B)**, and both **(C)**. The AUCs of CYFRA21-1 combined with miR-5684 **(D)**, miR-125b-5p **(E)**, and both **(F)**. The AUCs of CEA and CYFRA21-1 combined with miR-5684 **(G)**, miR-125b-5p **(H)**, and both **(I)**.

Carcinoembryonic antigen (CEA) is a pan-tumor prognostic marker and is recommended for diagnosing NSCLC (22). As shown in **Figures 6A, B**, miR-5684 or miR-125b-5p elevated the AUC of CEA significantly, from 0.791 to 0.85 or 0.839. As expected, combining all three markers increased the AUC value to 0.877 (95% CI, 0.848–0.906), and the sensitivity and specificity to 69.5 and 90.1% respectively (**Figure 6C**).

The cytokeratin 19 fragment CYFRA21-1 is an established circulatory tumor marker, and showed an AUC of 0.751. The combination of miR-5684 or miR-125b-5p and CYFRA21-1 had a higher AUC of 0.837 or 0.813 (**Figures 6D, E**). The combination of all three had a higher AUC of 0.863 (**Figure 6F**). Interestingly, combining either exosomal miR-5684 or miR-125b-5p with CEA and CYFRA21-1 resulted in AUCs of 0.878 and 0.868, respectively (**Figures 6G, H****)**. Importantly, the highest AUC of 0.896 (95% CI, 0.869–0.923) was obtained with the combination of all of the above markers, and the sensitivity and specificity were correspondingly increased to 72.9 and 92.5% respectively (**Figure 6I**).

**Figure 7:**
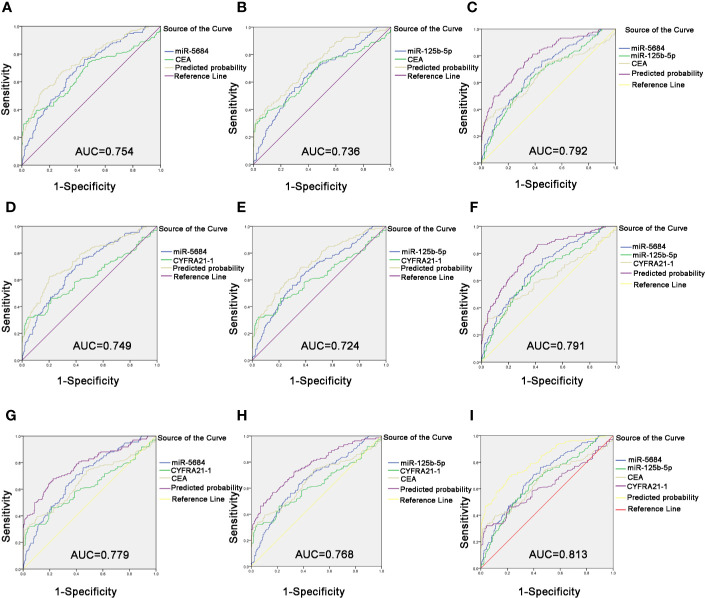
Improved diagnostic capacity of serum exosomal miRNAs combined with established tumor markers in early-stage NSCLC patients. The AUCs of CEA combined with miR-5684 **(A)**, miR-125b-5p **(B)**, and both **(C)**. The AUCs of CYFRA21-1 combined with miR-5684 **(D)**, miR-125b-5p **(E)**, and both **(F)**. The AUCs of CEA and CYFRA21-1 combined with miR-5684 **(G)**, miR-125b-5p **(H)**, and both **(I)**.

Likewise, the combination of CEA with serum exosomal miR-5684 (AUC = 0.754) or miR-125b-5p (AUC = 0.736) significantly improved the diagnostic efficiency of early-stage NSCLC compared to CEA alone (**Figures 7A, B**). The combination of all three further improved the diagnostic accuracy of early-stage NSCLC patients (AUC = 0.792 (95% CI, 0.745–0.839; 75.4% sensitivity and 68% specificity) compared to healthy donors (**Figure 7C**). Similarly, the combination of miR-5684 or miR-125b-5 with CYFRA21-1 resulted in higher AUC (0.749 and 0.724, respectively) compared to CYFRA21-1 alone (**Figures 7D, E**). As expected, miR-5684 and miR-125b-5p combined with CYFRA21-1 had a higher AUC of 0.791 for early stage NSCLC versus healthy subjects (**Figure 7F**). Furthermore, combination of miR-5684 or miR-125b-5p with CEA and CYFRA21-1 had an AUC of 0.779 and 0.768 (**Figures 7G, H**) respectively, whereas the AUC of all four markers was 0.813 (95% CI, 0.767–0.858) with 69.4% sensitivity and 78.7% specificity (**Figure 7I**). Taken together, serum exosomal miR-5684 and miR-125b-5p can increase the diagnostic efficacy of established markers like CEA and CYFRA21-1 for NSCLC as well as its early stages.

### Serum Exosomal miRNAs are Associated with the Tumor Stage

**Figure 8:**
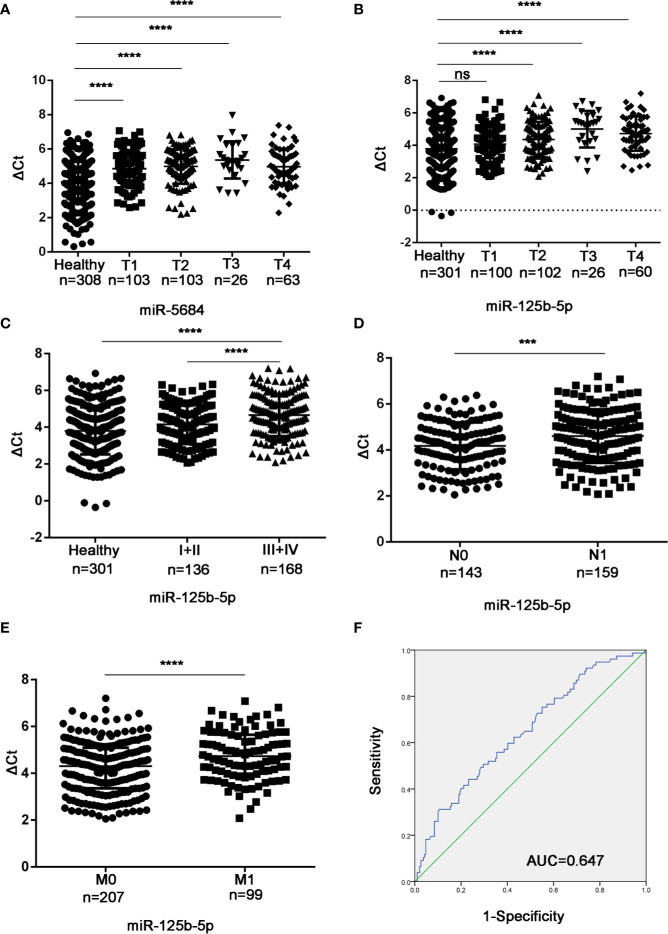
Association between the serum exosomal miRNAs and tumor stage. Expression levels of **(A)** miR-5684 and **(B)** miR-125b-5p in T and N stages patients. **(C)** Serum exosomal miR-125b-5p expression in early stage (I+II) and advanced stage (III+IV) patients. **(D, E)** Statistical association between miR-125b-5p and **(D)** lymph node metastasis and **(E)** distant metastasis. **(F)** The AUC of serum exosomal miR-125b-5p was 0.647 in metastatic patients relative to non-metastatic NSCLC patients. (***P < 0.001, ****P < 0.0001, ns, not significant).

Studies have shown that the plasma level of miR-125b-5p is related to the disease stage of the international staging system in multiple myeloma (23). In addition, miR-125b has been reported to be associated with the metastasis of liver cancer and breast cancer (18, 24, 25). To determine the biological relevance of the serum exosomal miRNAs in NSCLC, we analyzed the relationship between their expression levels and the T/N stages. As shown in **Figure 8A**, exosomal miR-5684 expression was significantly lower in the T1-T4 NSCLC patients compared to healthy honors, whereas that of exosomal miR-125b-5p was significantly lower in the patients at T2 and higher stages (**Figure 8B**). Furthermore, exosomal miR-125b-5p accurately discriminated the early stage (I+II) and advanced stage (III+IV) patients (**Figure 8C**), as well as the lymph node negative and lymph node positive groups (**Figure 8D**). In fact, high levels of exosomal miR-125b-5p was associated with significantly less lymphatic invasion (*P* = 0.0008) and distant metastasis (*P* < 0.0001, **Figure 8E**). Finally, serum exosomal miR-125b-5p exhibited a high diagnostic accuracy for metastatic versus non-metastatic patients with an AUC of 0.647, and sensitivity and specificity 50.6% and 70.4% respectively. Taken together, serum exosomal miRNAs are useful biomarkers for early diagnosis of NSCLC and for predicting metastasis.

### Role of Exosomal miR-125b-5p in Chemotherapy Response Assessment

Studies have shown that the expression level of miR-125b-5p correlates to the neoadjuvant chemotherapy response and prognosis of breast cancer (26, 27). In order to explore the relationship between miR-125b-5p and NSCLC chemotherapy response, 70 NSCLC patients who received first-line chemotherapy were added to our study. These patients’ clinical response was graded according to the RECIST guidelines (28). Response rate (RR) is defined as the proportion of patients with complete remission (CR) and partial remission (PR) that can be evaluated as the best response; disease control rate (DCR) is defined as CR, PR and stable disease (SD) Proportion of patients with the best response (29). RR to the first-line chemotherapy in miR-125b-5p was 42.9%. In addition, DCR with the first-line chemotherapy in the exosomal miR-125b-5p was 82.9% (**Table 3**).

**Table 3:** Response to the first chemotherapy in serum exosomal miR-125b-5p.

Gene	N	CR	PR	SD	PD	RR%	DCR%
miR-125b-5p	70	0	30	28	12	42.9	82.9

CR, complete response; PR, partial response; SD, stable disease; PD, progressive disease; RR, response rate; DCR, disease control rate.

**Figure 9:**
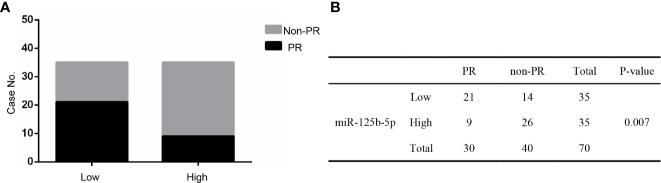
The relationship between serum exosomal miR-125b-5p and chemotherapeutic effect. **(A)** High expression of serum exosomal miR-125b-5p was correlated with favorable first chemotherapy response. **(B)** Response to the 1ST treatment in patients.

Furthermore, these patients were divided into PR and non-PR groups. Non-PR included SD and PD. The data showed that exosomal miR-125b-5p expression levels were lower in the non-PR group than that in PR groups (P=0.007, **Figures 9A, B**). Thus, serum exosomal miR-125b-5p is a potential biomarker for predicting the chemotherapeutic response.

### Validating the Prognostic Value of Exosomal miR-125b-5p in NSCLC

**Figure 10:**
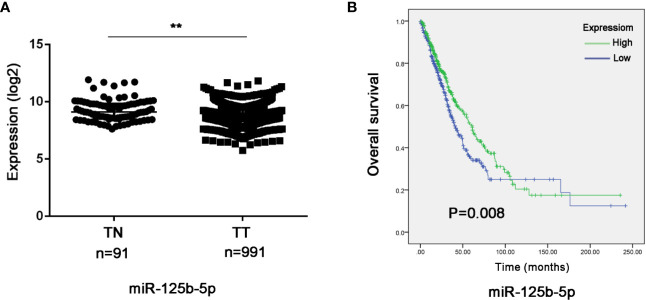
Validation of the prognostic value of serum exosomal miR-125b-5p in NSCLC. **(A)** Relative expression of miR-125b-5p in NSCLC compared to normal tissue in TCGA database. **(B)** Kaplan-Meier survival analysis of TGCA patients with low-expression and high-expression miR-125b-5p groups. TN, normal tissue; TT, tumor tissue. (**P < 0.01).

To further validate the prognostic relevance of miR-125b-5p in NSCLC, we analyzed the BCGSC miRNA profile data of 91 normal lung tissues and 991 NSCLC tissues from TCGA database. As shown in **Figure 10A**, miR-125b-5p expression was significantly decreased in the NSCLC tumors compared to the normal tissues (P=0.004). The patients (n=864) were then stratified into the miR-125b-5p high- or low-expression groups with median expression as the cut-off point. Patients expressing high levels of miR-125b-5p had significantly longer OS compared to the low-expression group (P =0.008; **Figure 10B**). Furthermore, univariate analysis showed that T stage (P =0.006), N stage (P=0.008), M stage (P=0.001), TNM stage (P < 0.0001) and miR-125b-5p expression levels (P=0.008) were significantly associated with the OS of NSCLC patients, whereas age (P=0.094), sex (P = 0.371) and pathological status (P = 0.501) had no significant influence. Multivariate analysis of the significant variables further indicated that only the miR-125b-5p expression level [HR=0.744, 95% confidence interval (CI): 0.600–0.923; P=0.007] was an independent prognostic factor for NSCLC (**Table 4**).

**Table 4:** The influence of different variables on OS for 864 patients with NSCLC analyzed by Cox proportional hazard model.

Variables	OS		
	Univariate	Multivariate	
	P	HR (95CI%)	P
Age	0.094		Not included
<67			
≥67			
Gender	0.371		Not included
Male			
Female			
T stage	0.006		0.925
T1		0.778(0.127-4.771)	0.786
T2		0.823(0.135-5.002)	0.833
T3		0.971(0.158-5.950)	0.974
T4		Reference	
N stage	0.008		0.655
pN0		Reference	
pN1		0.835(0.309-2.259)	0.655
M stage	0.001		0.829
M0		109.251(0-3.367E+20)	0.829
M1		Reference	
Stage	<0.0001		0.204
I		0.002(0-7.24E+15)	0.780
II		0.003(0-9.351E+15)	0.789
III		0.005(0-1.488E+16)	0.806
IV		Reference	
Pathology diagnosis	0.501		Not included
AC			
SCC			
miR-125b-5p	0.008	**0.744(0.600-0.923)**	**0.007**

Bold values: significant difference.

## Discussion

The poor prognosis of NSCLC patients is largely due to high recrudescence rates, reflecting the urgent needs of novel sensitive and specific biomarkers for early diagnosis. In current study, we screened out the exosomal miR-5684 and miR-125b-5p using microarray and validated their expression pattern in a large cohort, followed by the analysis for their diagnostic efficiency and predicting chemotherapy response assessment, thus providing the evidence of exosomal miR-5684 and miR-125b-5p as biomarkers for NSCLC.

MiR-125b has been reported previously in osteosarcoma (30), hematopoietic malignancies (31) and NSCLC (32). It is down-regulated in several malignancies and functions as a tumor suppressor in colorectal cancer, gallbladder cancer (33, 34), breast cancer (35) and esophageal squamous cell carcinoma (36), as well as suppresses bladder cancer development *via* inhibition of MALAT1 (32). Aberrant expression empowers miR-125b-5p the potential as the diagnostics and prognosis biomarkers. For instance, decreased serum levels of miR-125b-5p is associated with favorable prognosis in breast cancer (26, 27). MiR-125b-5p could serve as potential diagnostic biomarkers for ESCC and Multiple myeloma (23, 36). In current study, we demonstrated that these two exosomal miRNAs were down-regulated in NSCLC group compared to healthy group with an AUC of 0.793. Nevertheless, this is the first study to demonstrate a role of exosomal miR-5684 (https://www.genecards.org/cgibin/carddisp.pl?gene=MIR5684&keywords=miR-5684) in cancer.

Notably, downregulation of serum exosomal miR-125b-5p in NSCLC patients also correlated to chemoresistance and disease progression, and patients that achieved a PR expressed significantly higher levels of this miRNA, indicating its role as a predictor for monitoring chemotherapy response. Our findings indicated high expression of miR-125b-5p was correlated with “favorable” 1st chemotherapy response, thus providing a noninvasive marker to predict 1st chemotherapy response. Besides, we also evaluated the prognostic value of miRNA-125b-5p *via* analyzing the data from TCGA database. The higher expression of miR-125b-5p was associated with better OS, and lower level of miR-125b-5p level was associated with worse OS in lung cancer tissues. We assumed that exosomal miR-125b-5p could act as an independent predictor for NSCLC prognosis which needs further exploration.

The limitations of this study are the relatively small cohort size and lack of any mechanistic insights. Future studies ought to be conducted on larger cohorts to confirm our findings, and the mechanisms underlying the function of these exosomal miRNAs should also be elucidated through functional assays. In conclusion, serum exosomal miRNAs are promising non-invasive diagnostic and prognostic markers of NSCLC, and warrant clinical validation.

## Data Availability Statement

The raw data supporting the conclusions of this article will be made available by the authors, without undue reservation.

## Ethics Statement

The studies involving human participants were reviewed and approved by the Shandong Cancer Hospital Affiliated to Shandong First Medical University and Shandong Academy of Medical Sciences of committee. All subjects gave written informed consent in accordance with the Declaration of Helsinki.

## Author Contributions

SZ and XrS designed the experiments. ZZ carried out the experiments. YT wrote the manuscript and prepared the figures. XgS and LX contributed to analysis the experimental data. All authors contributed to the article and approved the submitted version.

## Funding

This work was supported by the National Natural Science Foundation of China (81773237, 81672104, 81972014), the Shandong Provincial Key Research and Development Program (2016GSF201146, 2017GSF18183 and 2017CXGC1207), Shandong Provincial Natural Science Foundation (ZR2019MH004 and ZR2019LZL016) and Science and technology development plans of Tai’an City (Project No. 2019NS213).

## Conflict of Interest

The authors declare that the research was conducted in the absence of any commercial or financial relationships that could be construed as a potential conflict of interest.

## Supplementary Material

Supplementary Figure 1 The expression levels of serum exosomal miRNAs in NSCLC patients and healthy donors. **(A)** miR-17-5p, **(B)** miR-9-5p, **(C)** miR-148-3p, and **(D)** miR-154-5p.
