# Supplementary figures and images for: Tumor-Derived Exosomal miRNAs as Diagnostic Biomarkers in Non-Small Cell Lung Cancer
Source: Front Oncol. 2020 Oct 14;10:560025. doi: 10.3389/fonc.2020.560025 (PMC7592397; doi:10.3389/fonc.2020.560025)

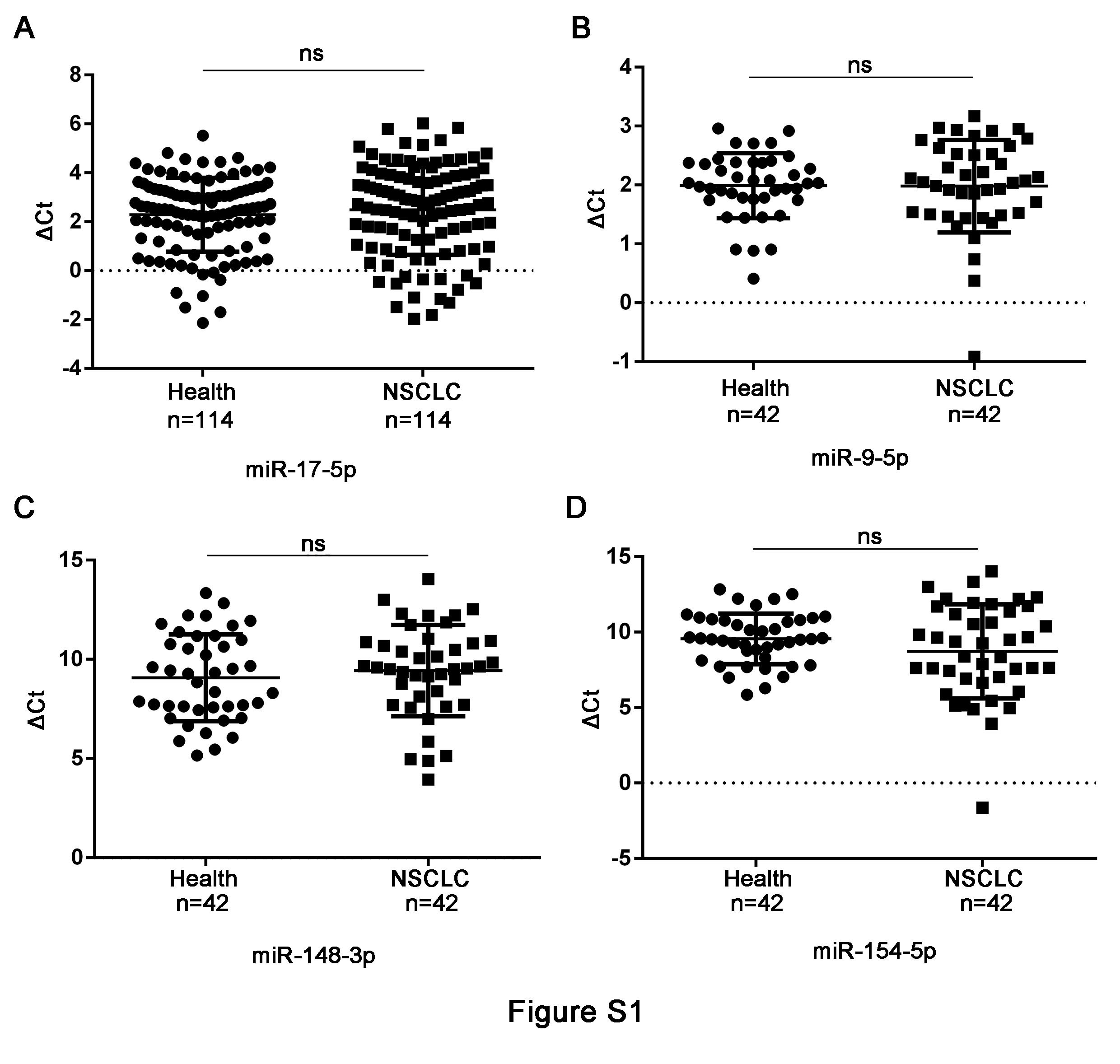

Supplement: Supplementary Figure 1 — The expression levels of serum exosomal miRNAs in NSCLC patients and healthy donors. (A) miR-17-5p, (B) miR-9-5p, (C) miR-148-3p, and (D) miR-154-5p. [file Image_1.jpeg]
