# Supplementary material for: Tumor-Derived Exosomal miRNAs as Diagnostic Biomarkers in Non-Small Cell Lung Cancer
Source: Front Oncol. 2020 Oct 14;10:560025. doi: 10.3389/fonc.2020.560025 (PMC7592397; doi:10.3389/fonc.2020.560025)
Supplement: Supplementary file 2 [file Table_1.docx]

**Table S1**. Relationship between early clinical characteristics and exosomal miR-5684, miR-125b-5p expression.

| **Characteristics** | | | **miR-5684** | | |  | | **miR-125b-5p** | | |
| --- | --- | --- | --- | --- | --- | --- | --- | --- | --- | --- |
|  | | **No. case** | | **Median** | ***P*-value** |  | **No. case** | | **Median** | ***P*-value** |
| Age (y) | ≤62 | 78 | | 4.8970 | 0.583 |  | 76 | | 4.1181 | 0.503 |
|  | >62 | 68 | | 4.8057 |  |  | 69 | | 4.2354 |  |
| Gender | Male | 69 | | 4.9751 | 0.167 |  | 71 | | 4.0576 | 0.191 |
|  | Female | 77 | | 4.7464 |  |  | 74 | | 4.2855 |  |
| Smoking | Yes | 58 | | 4.8979 | 0.671 |  | 59 | | 4.0897 | 0.425 |
|  | No | 88 | | 4.8259 |  |  | 86 | | 4.2317 |  |
| Drinking | Yes | 37 | | 4.7192 | 0.342 |  | 38 | | 4.0988 | 0.602 |
|  | No | 109 | | 4.9004 |  |  | 107 | | 4.2006 |  |
| Pathology diagnosis | AC | 112 | | 4.8365 | 0.443 |  | 106 | | 4.1392 | 0.950 |
|  | SCC | 33 | | 4.9880 |  |  | 31 | | 4.1523 |  |

AC, adenocarcinoma; SCC, squamous cell carcinoma.

**Table S2.** The diagnostic values of exosomal miR-5684 and miR-125b-5p combined with or without CEA, CYFRA21-1 in NSCLC

| Diagnostic markers | AUC | 95%CI | Sensitivity | Specificity |
| --- | --- | --- | --- | --- |
| miR-5684 | 0.733 | 0.690-0.775 | 0.752 | 0.605 |
| miR-125b-5p | 0.700 | 0.655-0.745 | 0.624 | 0.7 |
| miR-5684 and miR-125b-5p | 0.793 | 0.755-0.831 | 0.827 | 0.621 |
| miR-5684 and CEA | 0.850 | 0.818-0.883 | 0.707 | 0.866 |
| miR-125b-5p and CEA | 0.839 | 0.805-0.873 | 0.662 | 0.881 |
| miR-5684, miR-125b-5p and CEA | 0.877 | 0.848-0.906 | 0.695 | 0.901 |
| miR-5684 and CYFRA21-1 | 0.837 | 0.803-0.871 | 0.759 | 0.783 |
| miR-125b-5p and CYFRA21-1 | 0.813 | 0.777-0.850 | 0.602 | 0.921 |
| miR-5684, miR-125b-5p and CYFRA21-1 | 0.863 | 0.832-0.894 | 0.714 | 0.854 |
| miR-5684, CEA and CYFRA21-1 | 0.878 | 0.847-0.908 | 0.726 | 0.901 |
| miR-125b-5p, CEA and CYFRA21-1 | 0.868 | 0.837-0.900 | 0.718 | 0.917 |
| miR-5684，miR-125b-5p，CEA and CYFRA21-1 | 0.896 | 0.869-0.923 | 0.729 | 0.925 |

**Table S3.** The diagnostic values of exosomal miR-5684 and miR-125b-5p combined with or without CEA, CYFRA21-1 in early-stage NSCLC

| Diagnostic markers | AUC | 95%CI | Sensitivity | Specificity |
| --- | --- | --- | --- | --- |
| miR-5684 | 0.696 | 0.642-0.750 | 0.761 | 0.549 |
| miR-125b-5p | 0.66 | 0.603-0.717 | 0.627 | 0.636 |
| miR-5684 and miR-125b-5p | 0.744 | 0.693-0.794 | 0.806 | 0.609 |
| miR-5684 and CEA | 0.754 | 0.703-0.806 | 0.530 | 0.858 |
| miR-125b-5p and CEA | 0.736 | 0.684-0.789 | 0.731 | 0.605 |
| miR-5684, miR-125b-5p and CEA | 0.792 | 0.745-0.839 | 0.754 | 0.68 |
| miR-5684 and CYFRA21-1 | 0.749 | 0.697-0.801 | 0.627 | 0.794 |
| miR-125b-5p and CYFRA21-1 | 0.724 | 0.67-0.777 | 0.604 | 0.731 |
| miR-5684, miR-125b-5p and CYFRA21-1 | 0.791 | 0.743-0.838 | 0.784 | 0.676 |
| miR-5684, CEA and CYFRA21-1 | 0.779 | 0.727-0.830 | 0.679 | 0.775 |
| miR-125b-5p, CEA and CYFRA21-1 | 0.768 | 0.717-0.819 | 0.567 | 0.834 |
| miR-5684，miR-125b-5p，CEA and CYFRA21-1 | 0.813 | 0.767-0.858 | 0.694 | 0.787 |
